# Antibacterial Properties of Ethacridine Lactate and Sulfmethoxazole Loaded Functionalized Graphene Oxide Nanocomposites

**DOI:** 10.3390/antibiotics12040755

**Published:** 2023-04-14

**Authors:** Tooba Jabri, Naveed Ahmed Khan, Zinb Makhlouf, Noor Akbar, Jasra Gul, Muhammad Raza Shah, Ruqaiyyah Siddiqui

**Affiliations:** 1 International Centre for Chemical and Biological Sciences, H.E.J. Research Institute of Chemistry, University of Karachi, Karachi 75270, Pakistan; 2 Department of Clinical Sciences, College of Medicine, University of Sharjah, Sharjah 27272, United Arab Emirates; 3 Department of Medical Biology, Faculty of Medicine, Istinye University, Istanbul 34010, Turkey; 4 College of Arts and Sciences, American University of Sharjah, Sharjah 26666, United Arab Emirates; 5 Research Institute of Medical & Health Sciences, University of Sharjah, University City, Sharjah 27272, United Arab Emirates

**Keywords:** antimicrobial resistance, graphene oxide, ethacridine lactate, sulfamethoxazole, glucosamine

## Abstract

The emergence of drug-resistant bacterial strains that reduce the effectiveness of antimicrobial agents has become a major ongoing health concern in recent years. It is therefore necessary to find new antibacterials with broad-spectrum activity against both Gram-positive and Gram-negative bacteria, and/or to use nanotechnology to boost the potency of already available medications. In this research, we examined the antibacterial efficacy of sulfamethoxazole and ethacridine lactate loaded two-dimensional glucosamine functionalized graphene-based nanocarriers against a range of bacterial isolates. Graphene oxide was first functionalized with glucosamine, which as a carbohydrate moiety can render hydrophilic and biocompatible characters to the GO surface, and subsequently loaded with ethacridine lactate and sulfamethoxazole. The resulting nanoformulations had distinct, controllable physiochemical properties. By analyzing the formulation using Fourier Transform Infrared Spectroscopy (FTIR), X-ray diffraction (PXRD), a thermogravimetric analysis (TGA), zetasizer, and a morphological analysis using Scanning Electron Microscopy and Atomic Force Microscopy, researchers were able to confirm the synthesis of nanocarriers. Both nanoformulations were tested against Gram-negative bacteria, including *Escherichia coli* K1, *Serratia marcescens*, *Pseudomonas aeruginosa*, *Salmonella enterica*, as well as Gram-positive bacteria, including *Bacillus cereus*, *Streptococcus pyogenes*, and *Streptococcus pneumoniae*. Importantly, ethacridine lactate and its nanoformulations exhibited significant antibacterial properties against all bacteria tested in this study. When tested for minimum inhibitory concentration (MIC), the results were remarkable and revealed that ethacridine lactate presented MIC_90_ at 9.7 µg/mL against *S. enteric*, and MIC_90_ at 6.2 µg/mL against *B. cereus*. Notably, ethacridine lactate and its nanoformulations showed limited toxicity effects against human cells using lactate dehydrogenase assays. Overall, the results revealed that ethacridine lactate and its nanoformulations possess antibacterial activities against various Gram-negative and Gram-positive bacteria and that nanotechnology can be employed for the targeted delivery of effective drugs without harming the host tissue.

## 1. Introduction

Antibiotic resistance is responsible for around 1.3 million deaths globally [1]. It is expected that the annual incidence of bacterial infections will reach ten million in the coming years, surpassing cancer [2]. This is no surprise as bacteria are evolving and/or gaining resistance against various antibiotic classes. For example, fluoroquinolones, aminoglycosides, monobactams, macrolides, cephalosporins, carbapenems, and penicillin are losing efficacy, as shown by drug-resistant bacteria, and there are increasing reports of antibiotic-resistant bacteria [3]. A survey of tap water from 25 cities of seven countries reports detecting 181 antibiotic-resistant genes [4]. Others report having detected airborne, antibiotic-resistant bacteria in farms, river water, and diseased pets [5,6,7]. Accordingly, there is a pressing need to discover antibacterial agents that can target various Gram-negative as well as Gram-positive bacteria, and/or enhance the efficacy of existing drugs. Recent research has concentrated on the development of nanoscale materials to increase the efficacy of antimicrobials. Carbon-based nanomaterials, fullerenes, carbon nanotubes (CNTs) (especially single-walled carbon nanotubes (SWCNTs)), and graphene oxide (GO) nanoparticles have drawn significant interest as potent antimicrobial agents. Their distinctive physiochemical characteristics, such as high surface area and chemical stability, enable the direct contact of microorganisms with carbon nanostructures, thereby seriously affecting their cellular membrane integrity, metabolic processes and morphology. Moreover, these carbon-based structures can be adapted for application in a pristine form or as nanocomposites. For the purpose of wastewater purification, the antibacterial properties of carbon nanotubes, fullerene, graphene, graphene oxide, and their nanocomposites have been studied [8,9]. Among them, graphene oxide, GO, a two-dimensional honeycomb lattice flat sheet, showed intriguing properties including biocompatibility, high surface to volume ratio, and high stability [10]. The development of graphene oxide nanomaterial for antimicrobial potential can be tailored with the modification of the functional groups (hydroxyl, carboxyl or epoxy) attached on its basal plane and edges, the incorporation of metal oxide nanoparticles at its surface, or the incorporation of antimicrobial agents. The underlying mechanisms of a GO-based nanocomposite are likely through the production of reactive oxygen species (ROS), leading to oxidative stress in microbial cells [11]. The conjugation of polydopamine nanoparticles with GO and ε-poly-L-lysine resulted in the disinfection of drug-resistant bacteria and the removal of harmful waterborne pollutants from the environmental samples [12]. The multifunctional architecture was used for effective separation, label-free SERS detection, and the eradication of methicillin-resistant *Staphylococcus aureus* (MRSA) due to the inclusion of antibacterial non-toxic biopolymer chitosan with GO [13]. Glucosamine (GLA) is an essential amino sugar and its conjugation tonanomaterial may improve the antimicrobial properties [14,15]. A previous report suggested that glucosamine-functionalized Ag nanoparticles enhanced the in vitro antibacterial inhibition, which facilitated the ability of the glucosamine-functionalized nanostructure to penetrate bacterial cell surfaces [14]. In addition, the antimicrobial agent ethacridine lactate (EL), also known as Acrinol or Rivanol, is an aromatic, organic, and highly soluble lactate salt of 2-ethoxy-6,9-diaminoacridine, commonly used as an antiseptic. It has been found to be effective against *Streptococcus* and *Staphylococcus,* while ineffective against *Pseudomonas* [16]. Its 0.1% solution is used as an antiseptic. The mode of action included microbial DNA binding and depletion by entering between the base pairs of the bacterial DNA [17,18]. Sulfamethoxazole (Sul) is a broad-spectrum antibiotic belonging to sulphonamides, which are structural analogues of p-amino benzoic acid which has a bacteriostatic effect [19]. It is used to treat urinary tract infections, prostatitis, and bronchitis. Sul inhibits the bacterial synthesis of tetrahydrofolic acid, a physiologically active form of folic acid. Additionally, it halts the synthesis of bacterial DNA, thymidine, and purines [20,21]. In this study, we tested the effects of ethacridine lactate and sulfamethoxazole against various bacterial isolates as well as the designed glucosamine-functionalized nanomaterials, obtained by the amide functionalization of glucosamine on a graphene oxide surface as a multifunctional nanocarrier to load EL and Sul. The insights of this study revealed that EL alone and a GO-based nanocarrier loaded with EL showed a high potency to eradicate various bacteria tested in this study without harming the human cells.

## 2. Results and Discussion

**Scheme 1:**
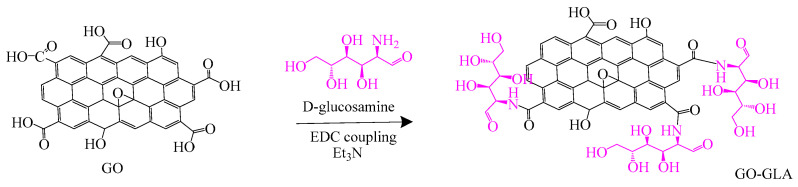
Synthesis of glucosamine-functionalized, graphene oxide nanoparticles (GO–GLA).

**Scheme 2:**
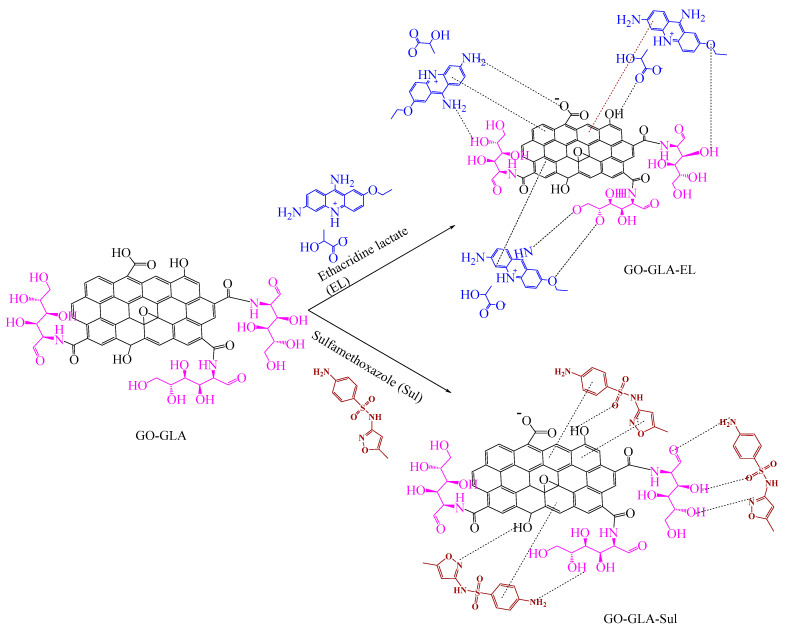
Antibacterial drugs (EL and Sul) loaded on GO–GLA nanocarriers.

Graphene oxide (GO) was successfully synthesized by an improved Hummer’s Method [22]. The synthesized GO was further functionalized to increase its stability, effectiveness, and specificity with glucosamine through EDC/NHS coupling via amide linkage as shown in Scheme 1. The resultant GO functionalized glucosamine was utilized as a nanocarrier, which is then loaded with two different drugs, EL and Sul, characterized and evaluated for its antibacterial and cytotoxicity activity as shown in Scheme 2. It is noteworthy that glucosamine functionalization takes place with the amide formation between the surface carboxylic moieties of GO and the amino function of glucosamine. With respect to the surface charges, pristine graphene oxide is negatively charged owing to the presence of a significant number of carboxylic moieties on its surface [23], which is also evident from its negative zeta potentials discussed further in this work. Sulfamethoxazole is generally neutral at pH = 7 [24], whereas ethacridine lactate, being a salt, is composed of ionic counterparts. The interaction between GO–GLA and the loaded drugs is strictly non-covalent, involving predominantly electrostatic interaction, ion-dipole, hydrogen bonding, and π–π interaction between various counterparts, as represented in the scheme.

### 2.1. FTIR Analysis

**Figure 1:**
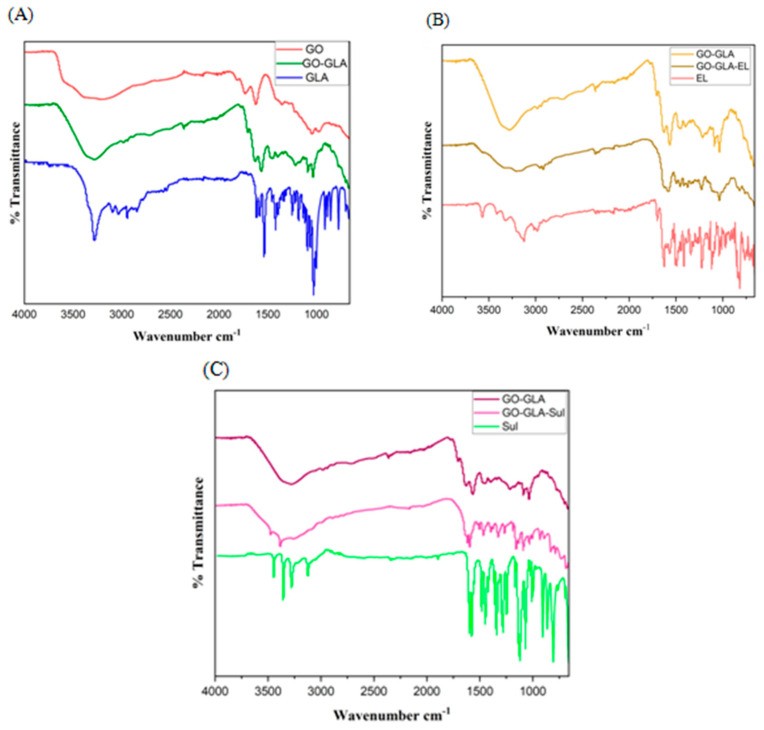
Fourier transform infrared analysis of NPs and nanoconjugates (**A**) FTIR spectrum of GO–GLA, (**B**) FTIR spectrum of GO–GLA–EL, (**C**) FTIR spectrum of GO–GLA–Sul.

FTIR spectroscopy of GO, GO–GLA, GO–GLA–EL, and GO–GLA–Sul was conducted to confirm the synthesis, modification, and drug loading of GO. In Figure 1A, the characteristic peaks of GO can be observed. At 3360 cm^−1^ a broad peak appeared due to the –OH vibrations. The peak at 1740 cm^−1^ is ascribed to the stretching vibration of –C=O fromthe carboxylic acid. The skeletal vibrations of the unoxidized graphitic spaces and the adsorbed water particles generate a peak at 1630 cm^−1^. The weak bands observed at 1220 cm^−1^ and 1050 cm^−1^ were assigned to the stretching vibrations of the C-O of alkoxy groups present at the edges of the graphene plane [25]. The FTIR spectrum of GO–GLA reveals the substantial structural changes as a result of the functionalization of GLA on the surface of the GO nanosheets. The band at 3283 cm^−1^ corresponds to the stretching vibration of N-H and O-H. The absorption band at 1627 cm^−1^ was observed due to the carbonyl stretching of the amide group. The appearance of a new peak at 1567 cm^−1^ is due to a secondary amine -NH bend. At 1214 cm^−1^ and 1088 cm^−1^ weak epoxy signals were observed [26]. Therefore, the spectrum shows the efficient conjugation of glucosamine with GO and forms a GO–GLA nanocarrier. The successfully synthesized GO–GLA nanocarrier was further loaded with EL and Sul. In Figure 1B the spectrum of GO–GLA–EL is depicted which exhibits the characteristic peaks of an ethacridine lactate band in the region of 3500–3100 cm^−1^ which is assigned to the asymmetric and symmetric stretching vibrations of primary aromatic amines. This region shows a broad band in GO–GLA–EL, corresponding to the merging of –NH stretching with the –OH of GO on account of the newly built non-covalent interaction between the oxygen rich functionalities of GO–GLA and EL. Moreover, the band appearing at 1650 cm^−1^ is attributed to the C=N of the acridine ring. The band at 1580 cm^−1^ corresponds to the COO- asymmetric stretch of the lactate anion and the C=C aromatic stretching vibrations [27,28]. The IR spectrum of the Sul loaded GO–GLA is illustrated in Figure 1C. The characteristic bands related to Sul were observed at 3480 cm^−1^, 3390 cm^−1^, and 3270 cm^−1^ and corresponds to the –NH group, and the asymmetric and symmetric vibration of SO_2_ appeared at 1470 cm^−1^. The peak at 1620 cm^−1^ and 1590 cm^−1^ appears due to the C=C of the aromatic ring and the stretching vibration of –NH amide, respectively [29]. The presence of the characteristic peaks of both drugs (EL and Sul) verifies the interaction of the drugs with the nanocomposite. It is worth noting that in comparison to the peaks of the various functionalities in the IR spectra of individual molecular entities, the IR spectra of the nanocomposite GO–GLA and the drug-loaded composites show a change in the intensity or position of the corresponding bands. This is understandably because of the newly formed covalent (GO–GLA) or non-covalent (hydrogen bonding, electrostatic, π-π) interactions within the fabricated composites.

### 2.2. Thermogravimetric Analysis (TGA)

**Figure 2:**
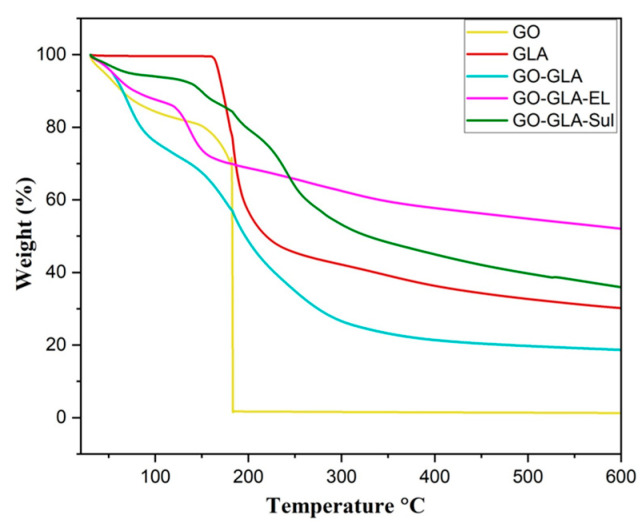
Thermogravimetric analysis of GO, GLA, GO–GLA, GO–GLA–EL and GO–GLA–Sul.

TGA analysis was performed to evaluate the thermal stability of GO, GLA-functionalized GO, and drug-loaded (EL and Sul) GO–GLA as depicted in Figure 2. The decomposition of the nanocomposite with temperature was investigated. The significant weight loss of GO was observed at >100 °C (8%) due to the evaporation of water molecule and moisture content, whereas as the mass loss from 100 °C to 200 °C (68%) attributed to the chemical decomposition of the oxygen of the acidic functional group and the hydroxyl group. The final loss above 200 °C resulted in the carbonyl decomposition [30]. The thermogram curve of GLA shows weight loss from 69 °C to 100 °C due to adsorbed water, and from approximately 160 °C to 240 °C rapid weight loss (48%) was observed. In the case of the GO–GLA nanocomposite, the TGA curve reveals decomposition at >100 °C (25%) attributed the moisture content, and the rapid weight loss observed at 150 °C, 230 °C, and 320 °C (54%). GO–GLA–EL and GO–GLA–Sul shows the total weight loss of ~36% and 45% in several steps that may be attributed to the water loss at <100 °C, followed by COOH decomposition, the detachment of glucosamine, and finally due to the possible detachment at 344 °C and 304 °C of EL and Sul, respectively.

### 2.3. Zeta Potential

Zeta potential is an important parameter to determine the stability and surface charge associated with nanocarrier systems. The variation in zeta potential depends on the chemical groups attached on the surface of nanoparticles [26]. The pristine GO shows the zeta potential of −26.8 mV. The zeta potential of GO–GLA observed an obvious decrease in the negative charge to −18.0 mV, indicating the GO loses carboxylic moiety when GLA is modified at the surface of the GO via the covalent interaction forming amide linkage. The EL- and Sul-loaded GO–GLA shows a slight increase in the negative zeta potential of GO–GLA to −22.6 mV and −22.2 mV, respectively, attributed to the conjugation of negatively charged drugs and indicating a successful loading of drugs on to the surface of GO–GLA.

### 2.4. Morphological Analysis

**Figure 3:**
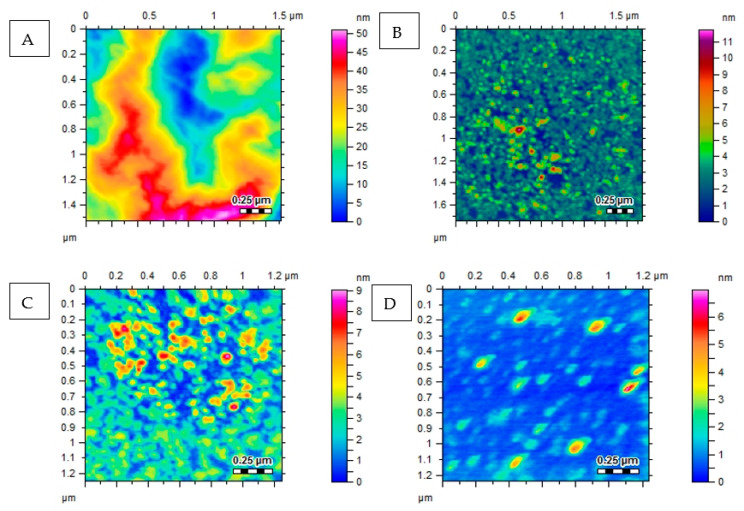
Representative images of the morphological analysis of (**A**) GO, (**B**) GO–GLA, (**C**) GO–GLA–EL, (**D**) GO–GLA–Sul using atomic force microscopy and the images.

**Figure 4:**
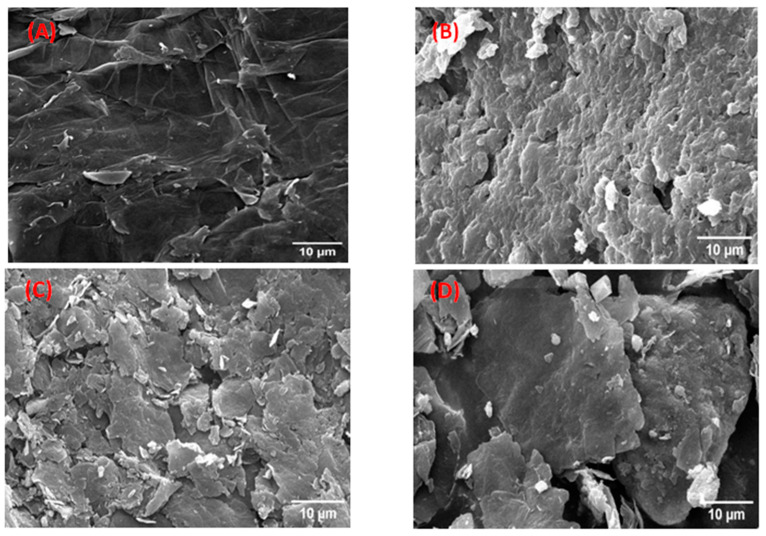
Scanning electron micrographs (SEM) of (**A**) GO, (**B**) GO–GLA, (**C**) GO–GLA–EL, (**D**) GO–GLA–Sul.

The surface morphology of the GO, GO–GLA, and drug-loaded GO–GLA was investigated by atomic force microscopy. The AFM and SEM image of GO shows the wrinkled structure and the twisting of layers attributed to the existence of epoxy, hydroxyl, and acidic functional groups on the surface of GO, which is caused by changes in the original structure of graphite sheets [31]. The surface morphology and wrinkled structure of virgin GO underwent evident alterations as a result of the attachment of glucosamine to it (Figure 1B). When treated with glucosamine, the GO–GLA composite shows a morphological change with noticeable roughness and tiny protuberances and ridges on the horizontal plate-like structure of GO as shown in both AFM (Figure 3B) and SEM micrographs (Figure 4B). The morphology of GO–GLA results from the existence of the amide bond between the amino groups of GLA and the carboxyl groups of GO, along with the hydrogen bonds between the hydroxyl and amino groups of CHI and the oxygen groups of GO. Further, the modification of GO–GLA by EL (Figure 3C and Figure 4C) and Sul (Figure 3D and Figure 4D) loading resulted in the further changes in the texture of GO due to drug molecules adhering to the surface of GO. The AFM images evidently show bright spots due to the attachment of conjugating drug molecules via non-covalent interactions with the surface functional groups of GO.

### 2.5. X-ray Diffraction Analysis

**Figure 5:**
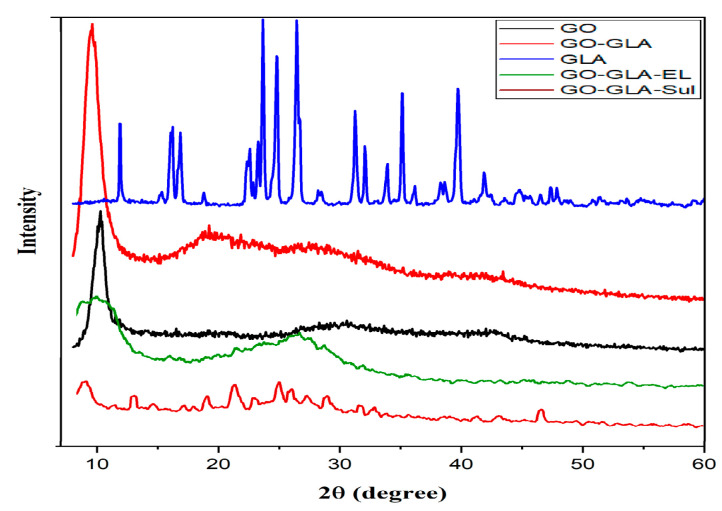
XRD diffraction pattern of GO, GLA, and GO–GLA, GO–GLA–EL and GO–GLA–Sul.

The X-ray diffractogram of the GO and modified GO (GO–GLA) nanocomposites are depicted in Figure 5. GO exhibits an intense and sharp characteristic diffraction peak at 2θ = 10.2°, with an interlayer d-spacing of 0.87 nm ascribed to the existence of oxygen-rich species of epoxy, hydroxyl and carboxyl at the surface edges of GO [32]. Meanwhile, the diffractogram of GLA exhibits sharp peaks corresponding to 2θ = 11.9°, 16.1°, 23.5°, 26.8°, and 31.2°. Additionally, the GO–GLA patterns lack the characteristic GLA peaks which results in the distorted broad diffraction peaks around 2θ = 19°, 28°, and 40°, showing an interlayer d-spacing of 0.46 nm, 0.32 nm, 0.225 nm. The modified GO–GLA manifests an amorphous nature as the GLA observes distortion and reduction in the interlayer d-spacing. The XRD patterns of GO–GLA–EL and GO–GLA–Sul manifest a further decrease in the crystallinity of the functionalized GO system, wherein the peak at 10.2° undergoes a further decrease in its intensity. This depicts that the drug-loaded composites are predominantly amorphous, with the existence of only small crystalline regions. This change in the crystallinity of the composites is presumably on account of the development of non-covalent interactions between the composites and the drug molecules.

### 2.6. Drug Loading

EL and Sul were loaded on the surface of the GO–GLA nanocarrier as an antibacterial drug. The entrapment efficiency of EL and Sul on GO–GLA was determined through free drug separation by centrifugation and its concentration was calculated by UV–vis spectrophotometer. The entrapment efficiency was found to be 89% and 83% for EL and Sul, respectively.

### 2.7. Graphene Oxide–GlucosamineDrugs Combination Demonstrated Remarkable Antibacterial Activities

**Figure 6:**
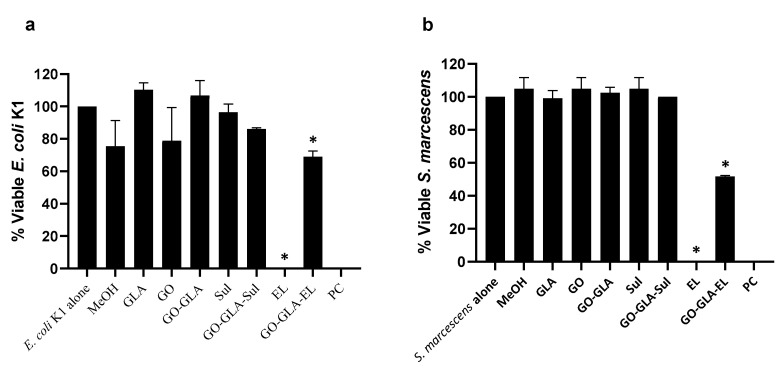

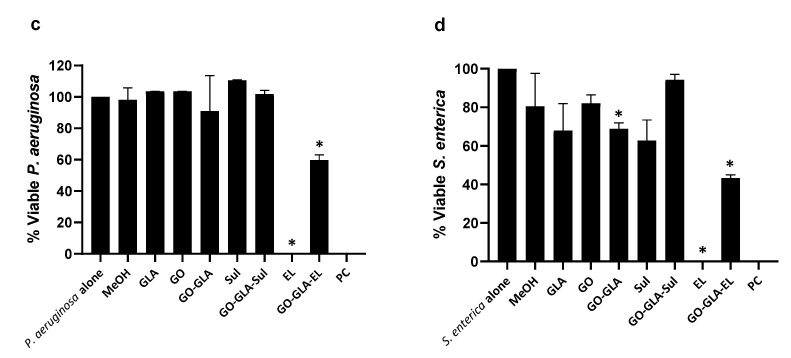
Antibacterial effects of drugs, NPs, and drug–NPs conjugates against Gram-negative bacteria. (**a**). antibacterial activity against *E. coli* K1, (**b**). antibacterial activity against *S. marcescens*, (**c**). antibacterial activity against *P. aeruginosa*, (**d**). antibacterial activity against *S. enterica*. All the experiments were performed three times in duplicate and the data is expressed as mean standard error and (*) denotes that *p* ≤ 0.05.

The antibacterial properties of NPs, drugs alone (ethacridine lactate and sulfamethoxazole), and the GO–GLA–drug nanoformulations were tested against a panel of Gram-positive and Gram-negative bacteria. The outcomes demonstrated that at 100 µg per mL dose, EL and drug conjugated NPs showed effective bactericidal effects against *E. coli* K1 and *S. marcescens* (*p* ≤ 0.05, Figure 6a,b). Remarkably, EL alone showed 100% bactericidal effects, while after the conjugation the nanocomposite revealed 31% and 49% antibacterial activity against *E. coli* K1 and *S. marcescens,* respectively (*p* ≤ 0.05, Figure 6a,b). Similarly, against *P. aeruginosa* and *S. enterica*, EL alone exhibited 100% killing while the EL-conjugate NPs showed 40% and 57% bactericidal properties (*p* ≤ 0.05, Figure 6c,d). The GO–GLA alone also demonstrated significant antibacterial activity, i.e., 31% (*p* ≤ 0.05, Figure 6d). All other drug(s), NPs, and their conjugates failed to show antibacterial effects against the Gram-negative bacteria tested in this study (Figure 6a–d). These findings are consistent with earlier studies by Petrikaite et al. who showed that ethacridine derivatives show promising activities against selected Gram-negative and Gram-positive bacteria in the range of 62.5–1000 µg per mL [33]. Regardless, our findings showed that the drug alone was more potent than the drug–NPs. This is possibly due to the slow release of the drugs in their nanoformulations, while the drugs alone exhibit direct effects on the bacteria tested. Overall, the results showed that EL exhibits potent antibacterial properties. The fact that ethacridine lactate alone exhibits potent antibacterial properties suggests that the observed effects are due to its antibacterial properties. Ethacridine lactate, an aromatic organic compound based on acridine, is a poly(ADP-ribose) glycohydrolase inhibitor whereby the compound binds to the DNA of bacteria, inhibiting the ability to synthesize DNA and RNA in the cell through interlocal incorporation between nucleotide base pairs in bacterial nucleic acid.

**Figure 7:**
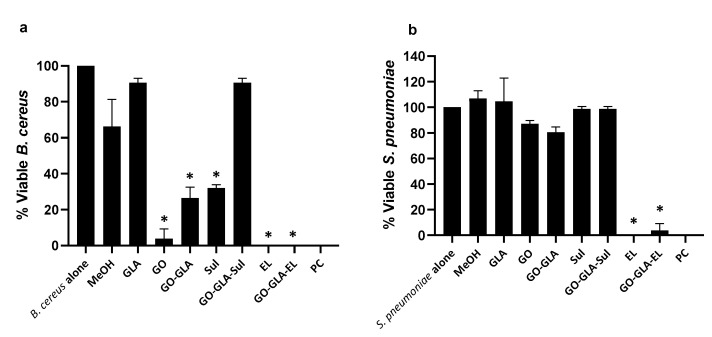

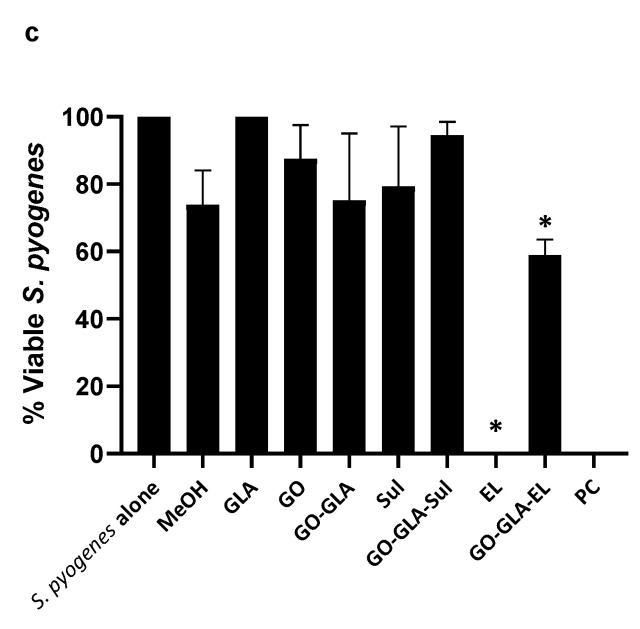
Bactericidal activity of drugs, NPs, and drug–NPs conjugates against Gram-positive superbugs. (**a**). when tested against *B. cereus*, (**b**) against *S. pneumoniae* and (**c**) against *S. pyogenes*. All the experiments were presented three times in duplicate and the data is expressed as mean standard error and (*) denotes that *p* ≤ 0.05.

**Figure 8:**
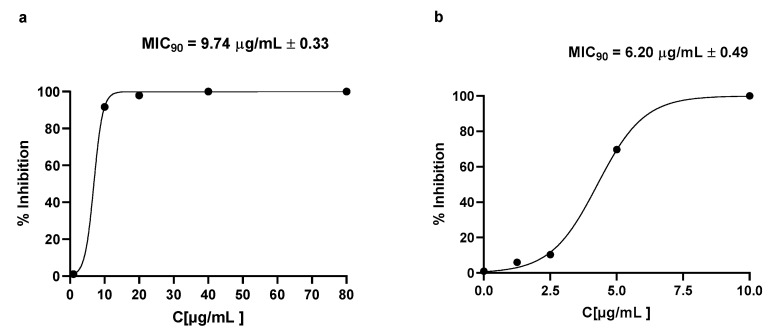
Minimum inhibitory concentration 90% of ethacridine lactate against (**a**) *S. enterica* and, (**b**) *B. cereus*.

When tested against Gram-positive, all the treatments except GLA and GO–GLA–Sul presented significant antibacterial properties against *B. cereus* (*p* ≤ 0.05, Figure 7a). At 100 µg per mL dose, EL alone showed 100% killing effects and GO, GO–GLA, and Sul showed 96%, 74% and 68% bactericidal activity towards *B. cereus,* respectively (*p* ≤ 0.05, Figure 7a). Among all the treatments, EL and its drug-conjugated counterpart inhibited 100% and 97% of *S. pneumoniae,* respectively, when compared to the negative control (*p* ≤ 0.05, Figure 7b). Whereas against *S. pyogenes*, EL showed constant 100% killing and GO–GLA–EL presented 41% antibacterial activity (*p* ≤ 0.05, Figure 7c). Results from MIC assays revealed that EL presented MIC_90_ at 9.74 µg/mL against *S. enteric* (Figure 8a). Against *B. cereus*, the EL showed MIC_90_ at 6.20 µg/mL (Figure 8b).

### 2.8. Drugs, Nanoparticles, and Drug–NPS Combination Revealed Limited Cytotoxic Effects against Human Cells

**Figure 9:**
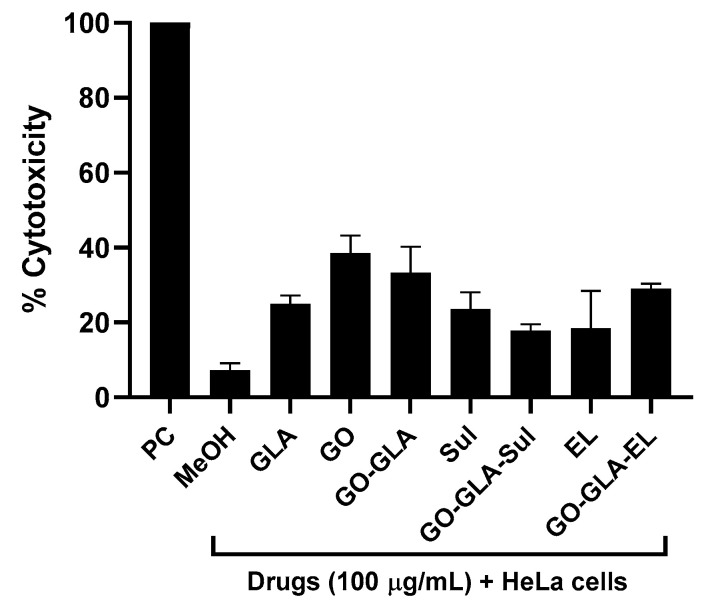
Cytotoxicity profiling of drugs, NPs, and their nanoconjugates against human cells. Note that the compounds/nanoformulations indicated minimal cytotoxicity towards human cells.

For cytotoxicity analysis, LDH assays were performed and the results revealed that all the treatments demonstrated limited cytotoxicity except graphene oxide and graphene oxide–glucosamine that showed moderate cytotoxicity against HeLa cells (Figure 9). The GO alone revealed 38% while the GO–GLA presented 33% of cytotoxic effects (Figure 9).

## 3. Materials and Methods

Graphite was purchased from AVONCHEM; glucosamine (GLA) was purchased from Merck; 1-ethyl-3-[3-(dimethylamino)propyl] carbodiimide hydrochloride (EDC) was purchased from Ambeed; triethyl amine (Et_3_N), sulfuric acid (H_2_SO_4_- 98%), and hydrochloric acid (HCl) were obtained from BDH laboratories; KMnO_4_ was used of analytical grade; hydrogen peroxide H_2_O_2_ (35%) was purchased from Scharlau chemicals; phosphoric acid, ethanol, and diethyl ether were purchased from Sigma Aldrich (St. Louis, MO, USA); and ethacridine lactate (EL) and sulfamethoxazole (Sul) were kind gifts from the local pharma. All these compounds were prepared and their working concentrations were used in different assays.

### 3.1. Synthesis of Graphene Oxide (GO)

The graphene oxide was prepared using the improved Hummer’s method reported earlier. Briefly, a 9:1 mixture of H_2_SO_4_:H_3_PO_4_ was added in graphite (3 g, 1 equiv) and stirred for 10 min, followed by the addition of KMnO_4_ (18.0 g, 6 equiv), which resulted in a slight exothermic to 35–40 °C. The reaction was then agitated for 12 h while being heated to 50 °C. The reaction mixture was poured onto ice with the dropwise addition of 30% H_2_O_2_ (3 mL) after being cooled at room temperature and the color changed to yellow. The solution was centrifuged at 4000× *g* for 1 h and the supernatant was decanted. The leftover solid residue was washed sequentially with water (300 mL), 30% HCl (300 mL × 2), and followed by ethanol (×2). In each step of washing, the solution was centrifuged at 4000× *g* for 1 h and the supernatant was discarded. The obtained solid material after successive washing was coagulated with ether and filtered and the residue was vacuum dried overnight at an ambient temperature. The GO synthesized from improved Hummer’s method was sonicated to yield nano graphene oxide.

### 3.2. Synthesis of Glucosamine Functionalized Graphene Oxide Nanoparticle (GO–GLA)

The functionalization of GO by GLA was achieved through the amidation process via EDC coupling. Briefly, GO (1 mg/mL, 50 mL) was sonicated to obtain the homogeneous suspension for 1 h, followed by the addition of EDC (250 mg) and NHS (150 mg) and stirred overnight to activate the carboxyl group. Then, GLA (250 mg) was added along with Et_3_N (1.1 equiv, with respect to GLA) and stirred overnight at an ambient temperature. Finally, the reaction mixture was centrifuged and then the residual was washed with water to remove the unreacted GLA. The product GO–GLA nanoparticles were lyophilized and stored for further use.

### 3.3. EL Loaded GO–GLA and Sul Loaded GO–GLA

Ethacridine lactate (EL) and Sulfamethoxazole (Sul) were loaded onto a GO–GLA nanocarrier as a drug delivery system. In general, EL (1 mg/mL) was dissolved in water and added to a GO–GLA solution stirred for 24 h at room temperature. Similarly, Sul, (1 mg/mL) dissolved in 1:1 water and methanol, was added in a GO–GLA solution stirred for 24 h at room temperature. Both drug-loaded solutions were centrifuged to remove the unbound drug. The free drug in supernatant was analyzed spectrophotometrically at 271 nm and 269 nm for EL and Sul, respectively. The drug loading efficiency was evaluated as follows: Drug loading efficiency = (total amount of drug − unbound drug)/Total amount of drug × 100.

### 3.4. Characterization Using FTIR

The FTIR analysis of samples was determined to investigate the possible interaction using ThermoNicolet’s Nicolet 6700 FTIR (Thermo Electron Corporation, Madison, WI, USA) spectrometer, equipped with a deutriated triglycine sulfate (DTGS) detector and connected to the OMNIC operating system software, to record the FTIR spectra. At room temperature, the samples were put on a ZnSe plate in direct contact with attenuated total reflectance (ATR). FTIR spectra were recorded at the range of 4000–650 cm^−1^ by adding 16 scans at the resolution of 4 cm^−1^.

### 3.5. Morphological Analysis

The morphological analysis of GO, GO–GLA, GO–GLA–EL, and GO–GLA–Sul was scanned through atomic force microscopy (5500, Agilent, Santa Clara, CA, USA). Nanocomposite sample solutions were diluted and a drop was casted on the mica side, air dried, and mounted on a microscope. The images were scanned in non-contact mode. Furthermore, the morphology of the nanocomposites was studied using a scanning electron microscope (SEM, model JSM-6380A, JEOL, Tokyo, Japan). Samples were placed in the metallic stub sputtered with gold and images were scanned under a microscope.

### 3.6. Zeta Potential

The zeta potential of nanoformulations was evaluated through Zetasizer (ZS-90 Malvern, UK). For analysis, the samples were dispersed in deionized water, poured in a cuvette, and measurements were recorded using dip cell in triplicates.

### 3.7. Thermogravimetric Analysis (TGA)

The thermal stability of prepared samples was analyzed using a thermogravimetric analysis (TGA) TA instrument SDT650. The samples were screened with a thermal ramp over a heating range of 25 °C–800 °C at a heating rate of 15.0 °C/min in a nitrogen atmosphere.

### 3.8. PXRD Analysis

X-ray diffraction (XRD) analysis was performed to investigate prepared GO and GO–GLA nanocomposites on an Xpert pro, PANaltyical diffractometer with Cu Kα (λ = 1.5418 Å).

### 3.9. Bacteria Used in This Study

Several bacteria were used in this study (Table 1). Bacterial isolates were purchased from American Type Culture Collection (ATCC) and Microbial Culture Collection (MTCC). They include *E. coli* K1, *Serratia marcescens*, *Salmonella enterica*, *Pseudomonas aeruginosa*, *Bacillus cereus*, *Streptococcus pyogenes,* and *Streptococcus pneumoniae*. Prior to the experiments, these bacteria were cultured overnight at 37 °C in nutrient broth [34].

### 3.10. Cultures of Henrietta Lacks (HeLa) Cervical Cancer Cells

Hela cells (ATCC CCL-2) were purchased from American Type Culture Collection and were grown in Roswell Park Memorial Institute (RPMI), complemented with 1% of Penicillin-Streptomycin, 10% Foetal bovine serum (FBS), 1% non-essential amino acid, and vitamins. The cells were cultured in a T-75 cm^3^ culture flask and maintained at 37 °C in a humidified incubator with 5% CO_2_. When 80–90% confluency was attained, the HeLa cells monolayer was trypsinized with Trypsin-EDTA for 5 min and cells were obtained. The culture containing HeLa cells was centrifuged and pellet was re-suspended in serum-free RPMI. Finally, the cells were counted by a haemocytometer and 1 × 10^4^ cell/well were seeded in 96-well plate and used for cytotoxicity assays.

### 3.11. Bactericidal Assays

Antibacterial assays were carried out to determine the antibacterial efficacy of the drug alone (EL and Sul), nanoparticle (NPs) (GO–GLA) alone, and drug–NPs conjugate (GO–GLA–Sul and GO–GLA–EL) against selected Gram-positive and Gram-negative bacteria [20]. In brief, the overnight grown bacterial culture optical density (OD) was adjusted to 0.22 at a 595 nm wavelength using spectrophotometer. This OD is equivalent to 1 × 10^8^ bacterial colony forming unit (c.f.u) per mL. A total of 10 µL of bacterial culture (equivalent to 1 × 10^6^ c.f.u/mL) was incubated with 100 µg/mL of drug, NPs, and drug–NPs conjugates for 2 h at 37 °C. For positive and negative controls, bacteria were incubated with Gentamicin and RPMI alone. After this incubation, bacterial cultures were ten-fold serially diluted and subsequently cultured on nutrient agar plates. The plates were incubated for overnight at 37 °C. Finally, viable bacterial colonies were counted and bacterial c.f.u/mL was recorded. In some of the experiments, various doses of the EL and drug–NPs conjugates (GO–GLA–EL) were tested against *S. enterica* and *B. cereus*. Against *S. enterica*, 10 µg/mL, 20 µg/mL, 40 µg/mL, and 80 µg/mL were tested while 1.25 µg/mL, 2.5 µg/mL, 5 µg/mL, and 10 µg/mL were tested against *B. cereus* to evaluate their minimum inhibitory concentration 90% (MIC90) [35].

### 3.12. Cytotoxicity Assays

Lactate dehydrogenase (LDH) experiments were carried out using human cell lines to ascertain the host cell cytotoxicity consequences of different medication [36]. Briefly, in a 96-well plate, a monolayer of HeLa cells was exposed to 100 µg/mL of various drugs and drug–NPs, and the plate was then kept for 24 h at 37 °C under 5% CO_2_ as well as 95% moisture. After this incubation, the supernatants were collected from each well and centrifuged to remove cellular debris and then cytotoxic effects were determined by estimating the amount of lactate dehydrogenase released from HBMEC using a Cytotoxicity Detection kit (Roche Applied Sciences, Penzberg, Germany). The assay involves the use of LDH to reduce NAD^+^ to NADH + H^+^ by oxidation of lactate to pyruvate. Then 2H are transferred from NADH + H^+^ to yellow tetrazolium salt INT [2-(4-iodophenyl)-3-(4-nitrophenyl)-5-phenyltetrazolium chloride] by a catalyst (diaphorase). An increase in the amount of dead or plasma membrane-damaged cells results in an increase of LDH enzyme release in the culture supernatant. This increase in the amount of enzyme activity in the supernatant directly correlates to the amount of formazan formed during the incubation time. Therefore, the amount of color formed in the assay is proportional to the number of lysed cells. The percentage of LDH release was detected as follows: (sample value−control value/total LDH release−control value × 100 = % cytotoxicity). As a positive and negative control, a cell monolayer treated using 0.1% Triton X-100 and RPMI alone, respectively, were used.

### 3.13. Statistical Analysis

Graph Pad Prism version 8.0.2 (GraphPad Software; San Diego, CA, USA) was used for all statistical analysis and visualizations. A *t*-test (two sample *t*-test, two-tailed distribution) was used to compare all the treatments with corresponding controls. The information is shown as the average standard deviation of multiple replicated experiments.

## 4. Conclusions

For the first time in this study, we have successfully synthesized graphene oxide-based nanocarriers functionalized with glucosamine and loaded with an antimicrobial agent. The successful synthesis of a nanocarrier was characterized by FTIR, XRD, TGA, SEM, and AFM analysis. When tested alone, ethacridine and sulfamethoxazole showed remarkable antibacterial effects against bacterial isolates tested, which is in agreement with the literature reports. However, their conjugation with GO–GLA resulted in potent but reduced antibacterial properties. A possible explanation is the targeted but reduced uptake of the drug nanoformulations by bacterial isolates tested in this study. Overall, these findings are promising and indicate that the conjugation of effective drugs, such as ethacridine, using nanotechnology holds promise for the targeted delivery of antibacterials without affecting the host tissue.

## Figures and Tables

**Table 1:** Bacterial species used in this study.

Bacteria	Strain
*Escherichia coli* K1	MTCC 710859 (clinical isolate)
*Serratia marcescens*	MTTC 13880 (clinical isolate)
*Pseudomonas aeruginosa*	ATCC 10145 (clinical isolate)
*Salmonella enterica*	ATTC 14028 (clinical isolate)
*Bacillus cereus*	MTCC 131621 (clinical isolate)
*Streptococcus pneumoniae*	ATCC 33400 (clinical isolate)
*Streptococcus pyogenes*	ATCC 12344 (clinical isolate)

## Data Availability

The data presented in this study are available on request from the corresponding author.
